# A CRISPR monkey model unravels a unique function of PINK1 in primate brains

**DOI:** 10.1186/s13024-019-0321-9

**Published:** 2019-05-02

**Authors:** Weili Yang, Shihua Li, Xiao-Jiang Li

**Affiliations:** 1 Guangdong-Hongkong-Macau Institute of CNS Regeneration, Ministry of Education CNS Regeneration Collaborative Joint Laboratory, Jinan University, Guangzhou, China; 2 Department of Human Genetics, Emory University School of Medicine, Atlanta, GA USA

**Keywords:** Parkinson’s disease, CRISPR/Cas9, Gene targeting, Primates, Neurodegeneration

## Abstract

Genetically modified rodent models have been valuable for investigating the pathogenesis of neurodegenerative diseases such as Parkinson’s disease (PD). Based on the fact that mutations in the *PINK1* gene cause autosomal recessive juvenile parkinsonism, a number of mouse models with deletion of the *PINK1* gene were generated. However, these *PINK1* knockout mouse models fail to recapitulate the selective and overt neurodegeneration seen in PD patient brains. Recently, we generated a non-human primate model with *PINK1* deletion using CRISPR/Cas9. This monkey model shows robust neurodegeneration in various brain regions, different from late-onset neurodegeneration in PD patients. Because of the limited pathological data available from humans carrying PINK1 mutations, the PINK1 mutant monkeys provide us with an important animal model to discuss the unique function of PINK1 that is essential for neuronal survival in primate brains. We also propose that the impairment of this unique function by *PINK1* mutations in humans may account for the age-dependent and progressive neurodegeneration.

## Background

Mutations in PTEN induced putative kinase 1 (*PINK1)*, a gene encoding a mitochondrial serine/threonine kinase, lead to autosomal recessive Parkinson’s disease (PD) [1]. A large body of in vitro findings have revealed that PINK1 is recruited to damaged mitochondria, leading to phosphorylation of Parkin and ubiquitin to eliminate aged and dysfunctional mitochondria via autophagy, a process called mitophagy that controls the quality of mitochondria [1, 2]. However, current *PINK1* knockout (KO) animal models have not validated these important in vitro findings [3, 4] or recapitulated selective and overt neurodegeneration seen in PD [5, 6]. Recently, we generated a *PINK1* KO monkey model using CRISPR/Cas9 and found that this model shows robust neurodegeneration in the cortex, striatum, and substantia nigra [7]. This finding raises immediate issues of why the monkey brain can show neurodegeneration when *PINK1* is knocked out and why this severe neurodegeneration is different from the age-dependent and progressive neurodegeneration in paitents with *PINK1* mutations.

## Main text

**Fig. 1:**
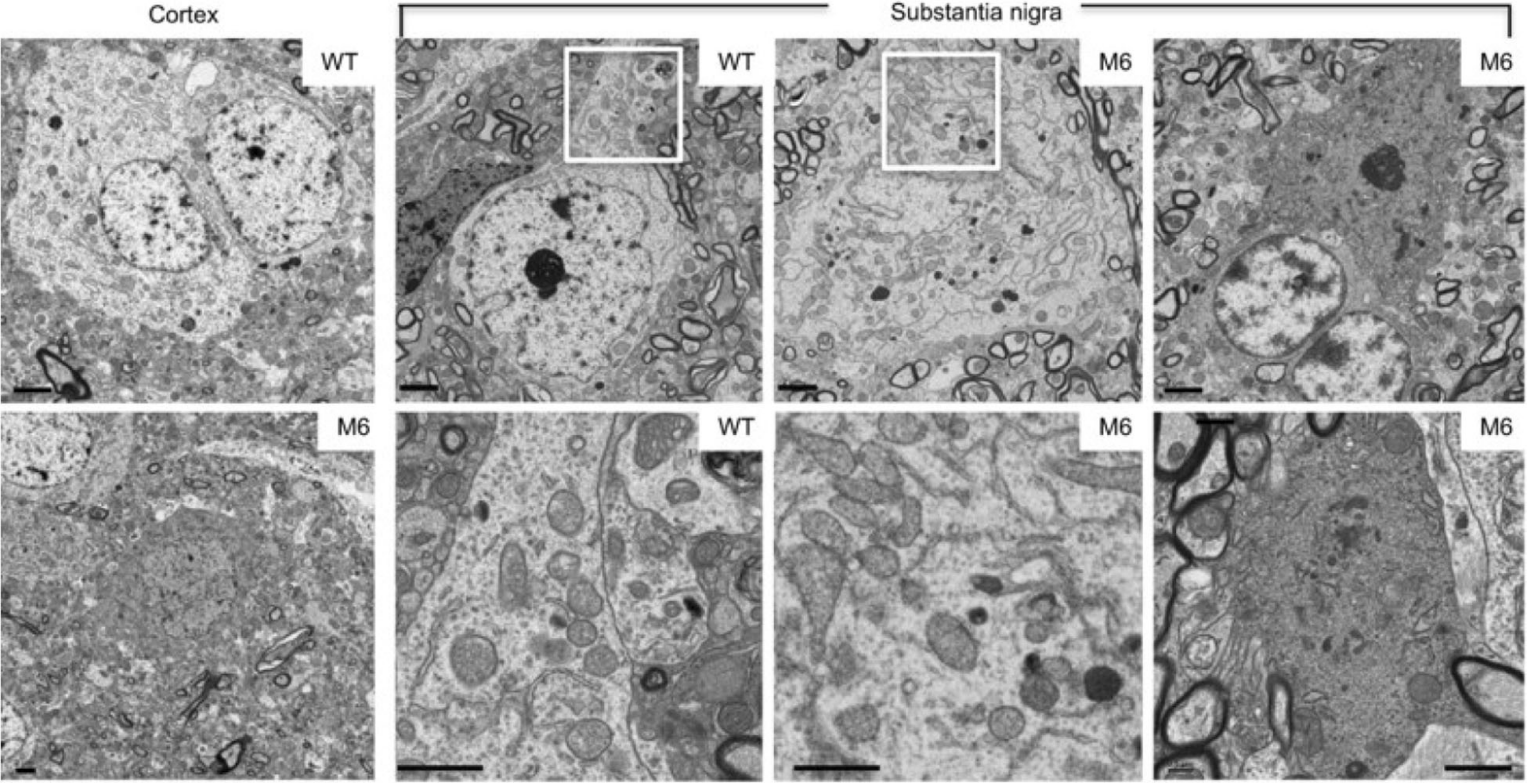
Neurodegeneration in *PINK1* mutant monkey brain. Electron microscopy revealed degenerated cells in the cortex and substantia nigra of a 3-year-old monkey (M6) in which the *PINK1* gene was targeted by CRISPR/Cas9 at one-cell embryo stage. Degenerated neurons show electron-dense cytoplasm with no clear organelles and no identifiable nuclear membrane, which are more obvious in dark neurons in the substantia nigra. The controls are the brain regions in a 3-year old male wild type (WT) monkey. The enlarged micrographs beneath the corresponding WT and M6 images are from their boxed areas and show that mitochondria morphology is indistinguishable between WT and M6 neurons. Scale bars: 2 μm. The figures are from authors’ publication [7]

Two important factors need to be considered when explaining the above issues. One is that species differences in genomics, anatomy, physiology may account for differential neuropathology in *PINK1* KO animals. It has been thought that short life span of rodents may not allow for developing age-dependent neurodegeneration, which often takes decades to occur in human brains. However, *PINK1* KO-mediated neurodegeneration does not appear to relate to the life spans of different species, because removing *PINK1* in Drosophila can lead to neurodegeneration, though massive muscle degeneration also occurs [8]. Great efforts have been taken to establish rodent models with PINK1 deletion. Only a rat model of *PINK1* KO was reported to show the selective reduction of tyrosine hydroxylase staining in the substantia nigra [9], but ultrastructural evidence of neurodegeneration in *Pink1* KO rodent models is still lacking. In the CRISPR/Cas9 targeted monkey, electron microscopy revealed degenerated cells in the cortex and substantia nigra. Degenerated neurons are dark neurons or show electron-dense cytoplasm with no clear organelles and no identifiable nuclear membrane (Fig. 1). However, in the *Pink1* KO mice that have a large deletion of the *PINK1* gene as CRISPR/Cas9 targeted monkeys, no evidence was provided to show that knocking out PINK1 could specifically eliminate the expression of PINK1 at the protein level and cause obvious neurodegeneration [5]. This paradoxical phenomenon was then explained by the very low level of PINK1 in rodents, which could only be detected by immunoprecipitation [10]. In contrast, the primate brains (monkey and human) show the abundant expression of PINK1 at the protein level [7]. Such differences of PINK1 expression in rodents and primates indicate that the abundant expression of PINK1 should play a critical and unique role in the primate brains.

Although depleting the *PINK1* gene in the monkey brain leads to neurodegeneration, the severe neuronal loss in various monkey brain regions (Fig. 1) is different from the age-dependent degeneration in PD patient brains. Also, PD is more likely to affect neurons in the substantia nigra in humans. Thus, the second factor attributable to differential neuropathology may be the nature of *PINK1* mutations in the monkey model and humans, as various types of mutations can result in different effects on PINK1 expression or function. The majority of *PINK1* mutations in humans are homozygous point mutations in one exon of the *PINK1* gene with a few cases of heterozygous large deletions [1]. The single locus mutations and heterozygous deletion may cause a partial loss of PINK1 expression and heterogeneity in PINK1 deficiency, therefore leading to different ages of onset (ranging from 9 to 61 years) and varying degrees of phenotypes in patients [1, 11, 12]. On the other hand, CRISPR/Cas9-mediated mutations in the monkey model result in the large deletion due to targeting two different *PINK1* exons, and this extensive deletion can completely eliminate PINK1 expression and function, leading to the more severe phenotype as seen in the dead newborn monkeys [7]. Because of the lack of studies of the postmortem brains from patients with homozygous *PINK1* mutations, we do not know how the complete loss of PINK1 affects neuronal cells in different brain regions in humans. Using western blotting to assess PINK1 expression and PCR to evaluate the relative degree of the large *PINK1* deletion, however, we found that CRISPR/Cas9-mediated deletion of the *PINK1* gene is well correlated with the extent of loss of PINK1 and neuronal cells in newborn monkeys [7]. It is possible that in humans, the complete loss of PINK1 leads to lethality during early development, so that only those mutations for a partial loss of PINK1 function were identified. It is also likely that neurons in the substantial nigra in the adult primate brain may be more vulnerable to the partial loss of PINK1 function. Indeed, the mosaicism of CRISPR-Cas9-mediated mutations also led to live *PINK1* mutant monkeys that showed a partial decrease in PINK1 and less severe neurodegeneration [7].

Thus, the unique expression of PINK1 in the primate brains and complete deletion of *PINK1* via CRISPR/Cas9 unraveled a critical function of PINK1 for the neuronal survival in the primate brains and also suggest that the identified *PINK1* mutations in humans may partially affect this critical function to cause late-onset neurodegeneration.

## Conclusion

Identification of neurodegeneration in the *PINK1* KO primate brains suggests that large animal models can more closely recapitulate the pathology of neurodegenerative diseases. This idea is also supported by Huntington’s disease (HD) knock-in pig model, which shows selective medium spiny neuron degeneration as HD patient brains [13]. Similar to most of rodent models of different neurodegenerative diseases (AD and PD), genetically modified HD mouse models are unable to mimic the overt and selective neurodegeneration in their brains. Thus, large animals should be considered as alternative models to investigate neurodegenerative diseases. However, there are limitations in using large animal models for research due to their high costs, the prolonged breeding periods, and ethical concerns as well as more strict regulations. These limitations obviously pose difficulties for a broad research community to use large animal models and will still demand rodents as the most widely used animal models. In addition, mosaic mutations generated by CRISPR/Cas9 also lead to heterogeneity in genotypes and phenotypes, which may be avoided by using a recently developed technology for cloning monkeys [14]. It should also be pointed out that CRISPR/Cas9-mediated gene depletion enables revealing the critical function of the targeted gene in the primates. The additional evidence to support this idea is that the CRISPR/Cas9-mediated large deletion of the autism-associated *SHANK3* gene, a mutation that does not exist in humans, also causes neuronal loss in the monkey brain during early development [15].

The valuable information obtained from the current *PINK1* KO monkey models would help us to further understand the pathogenesis of PD. For example, it would promote the use of neuronal cells of human origins, such as neurons derived from human stem cells, to investigate the critical function of PINK1. One important issue is how PINK1 is differentially expressed in different species such as rodents and primates. Is PINK1 expression regulated at the transcription/ translation level, or does its protein stability vary in a species-dependent manner? Because PINK1 expression in the rodent brains is at a very low level and PINK1 expression in the human brain remains to be explored, the *PINK1* KO monkey would provide a rigorous control for examining the expression and distribution of PINK1 in the primate brain. Another issue is how PINK1 deficiency affects the survival of the primate neuronal cells; does loss of PINK1 affect cellular mitochondrial/mitophagy function or protein phosphorylation due to the deficiency of PINK1 kinase? Moreover, how do *PINK1* mutations found in humans impair the important function of PINK1 and whether there is accumulation of α-synuclein or other neurotoxic proteins? Thus, the findings from the *PINK1* KO monkey model open up a new avenue for understanding the mechanism of selective neuronal loss in PD, which would also help identify new therapeutic strategies or targets.

## Abbreviations

AD: Alzheimer’s disease
CRISPR/Cas9: Clustered regularly interspaced short palindromic repeats/CRISPR-associated protein 9
HD: Huntington’s disease
KO: knockout
PD: Parkinson’s disease
PINK1: PTEN-induced putative kinase 1
WT: wild-type
